# Fabrication and characterization of silicon wire solar cells having ZnO nanorod antireflection coating on Al-doped ZnO seed layer

**DOI:** 10.1186/1556-276X-7-29

**Published:** 2012-01-05

**Authors:** Seong-Ho Baek, Bum-Young Noh, Il-Kyu Park, Jae Hyun Kim

**Affiliations:** 1 Energy Research Division, Daegu Gyeongbuk Institute of Science & Technology (DGIST), 50-1, Sang-Ri, Hyeonpung-Myeon, Dalseong-gun, Daegu, 711-873, South Korea; 2 Department of Electronic Engineering, Yeungnam University (YU), 214-1, Dae-Dong, Gyeongsan-Si, Gyeongbuk, 712-749, South Korea

**Keywords:** silicon microwire, solar cells, ZnO nanorods, antireflection coating, Al-doped ZnO, atomic layer deposition

## Abstract

In this study, we have fabricated and characterized the silicon [Si] wire solar cells with conformal ZnO nanorod antireflection coating [ARC] grown on a Al-doped ZnO [AZO] seed layer. Vertically aligned Si wire arrays were fabricated by electrochemical etching and, the p-n junction was prepared by spin-on dopant diffusion method. Hydrothermal growth of the ZnO nanorods was followed by AZO film deposition on high aspect ratio Si microwire arrays by atomic layer deposition [ALD]. The introduction of an ALD-deposited AZO film on Si wire arrays not only helps to create the ZnO nanorod arrays, but also has a strong impact on the reduction of surface recombination. The reflectance spectra show that ZnO nanorods were used as an efficient ARC to enhance light absorption by multiple scattering. Also, from the current-voltage results, we found that the combination of the AZO film and ZnO nanorods on Si wire solar cells leads to an increased power conversion efficiency by more than 27% compared to the cells without it.

## Introduction

In recent decades, most commercial solar cells are based on crystalline silicon [c-Si], but there is increasing efforts on thin film solar cells (second generation) as well as third generation solar cells which require the use of nano/microstructures for high efficiency and low cost [1]. Three-dimensional Si has been attracting much attention for future applications in photovoltaic devices due to their superior properties [2-9]. Si wire-based solar cells have two major advantages relative to commercial crystalline and thin-film Si absorbers. First, p-n junctions in the radial direction enable minority carriers to drift only short distances to the junction region for efficient carrier collection. That means low grade Si raw materials can be utilized, and manufacturing cost will be lowered [2]. In addition, the enhanced light absorption by an ordered wire is attributed to the light-trapping effect to the incident light [3,4]. Moreover, a wire array transfer technique has been studied, which not only yields c-Si wires on a flexible substrate for photovoltaic applications, but also allows the c-Si wafer to be reused for further production of aligned wire arrays [7,8].

For the fabrication of Si nano/microstructures, a number of bottom-up methods have been developed, such as vapor-liquid-solid [VLS] growth [5-8], chemical vapor deposition [CVD] [9], and molecular beam epitaxy [10]. However, these growth processes have some disadvantages as they generally need high temperature and high vacuum or discharge toxic precursors. As an alternative top-down route, a few lithographic procedures, such as electron beam lithography [11], and reactive ion etching [RIE] [12] are widely used in Si-based fabrication processes, but they are expensive, time-consuming, and not suited for mass production of ordered nanostructures on a large scale. In contrast, electrochemical etching, together with pre-patterning in a lithographic step is one of the most successful approaches in fabricating a large number of wires with a low cost and simple process. Unlike the growth techniques, vertically well-aligned Si wire arrays are reproduced by electrochemical etching with uniform periodicity [13]. Also, the formed Si wires have smooth surfaces, unlike those formed by using deep RIE where surfaces are damaged and wavy.

Nevertheless, Si wire solar cells still face critical challenges such as relatively low cell efficiency and surface recombination losses. Here, we investigated two key factors for the Si wire solar cells in order to improve the cell performances: One is to use ZnO nanorods to increase power conversion efficiency by suppressing light reflection and increasing light scattering to the Si wire solar cells. The other is to use an Al-doped ZnO [AZO] layer to passivate the Si surface and to facilitate the nucleation of ZnO nanorods.

Recently, ZnO nanorods are regarded as an efficient antireflection coating [ARC] to take advantage of its good transparency, appropriate refractive index (*n *= 2), and ability to form textured coating via anisotropic growth [14,15]. Several methods have been developed to grow ZnO nanorods, such as VLS process [16], CVD [17], and a hydrothermal method [18]. Among them, the hydrothermal method has been regarded as a low-temperature process with a large area growth and high growth rate. ZnO nanorods with high crystal quality can be grown perpendicularly on any surface of the substrates using hydrothermal synthesis. In addition, the seed layer is also important for the growth of high-quality ZnO nanorods. Prior to ZnO nanorod growth, AZO thin films were grown on high aspect ratio Si microwire [SiMW] arrays as a seed layer by atomic layer deposition [ALD] system. We introduced the AZO thin film as a buffer layer facilitating the nucleation and alignment of ZnO nanorods because AZO thin films are attractive due to their good conductivity, high transparency, and relatively low cost [19-22]. Moreover, the AZO film was deposited on the surface of Si wire arrays to suppress the surface recombination and increase the carrier collection efficiency. The ALD technique is the best choice for constructing composite thin films extremely conformal to the structure of a high aspect ratio.

In this study, we report the fabrication of highly ordered SiMW solar cells with conformal ZnO nanorod ARC grown on an AZO seed layer. The wire arrays of a c-Si were fabricated by means of electrochemical etching combining photolithography for site-selective etching. To evaluate the cell performances, the p-n junction was prepared by a spin-on dopant [SOD] method. AZO films were prepared by the ALD process, and ZnO nanorods grown on the as-prepared AZO seed layer were synthesized using hydrothermal growth methods. The morphological, optical, and photovoltaic properties of the SiMW solar cells having ZnO ARC were also characterized.

### Experimental details

#### Formation of the SiMW arrays

SiMW arrays were prepared in p-type < 100 > Si wafers with a resistivity of 1 to approximately 10 Ω cm (a boron doping density of 10^15 ^to approximately 10^16 ^cm^-3^) by electrochemical etching method. In order to make vertical arrays of Si wire with a high aspect ratio, we prepared the wafer pieces as follows: (1) The lithographical pattern was prepared on a silicon oxide layer as a mask to obtain an ordered array of a 2-μm square pattern spaced at a distance of 2 μm. (2) Then, the samples were dipped in a potassium hydroxide etchant to make inverse-shaped pyramidal notches, which would act as the regions for concentrating an electrical bias. (3) Electrochemical etching was performed with a mixed solution of hydrofluoric [HF] acid, dimethyl sulfoxide, and deionized water [DIW] (HF: (CH_3_)_2_SO: H_2_O = 2:5:15, *v*/*v*), respectively. (4) A thin aluminum [Al] layer with a thickness of 150 nm was deposited on the backside of the wafer to produce an ohmic contact between the Si wafer and working electrode by direct current magnetron sputtering method. After that, the electrochemical etching system was operated under a constant current density mode of different biases in a Teflon bath. A platinum wire was used as a counter electrode, and the Si wafer with Al coating on the backside was placed on a Teflon bath as a working electrode. The sample area exposed to the electrolyte solution was approximately 2 cm^2^.

### AZO seed layer and ZnO nanorods growth

AZO thin films have been prepared by ALD technique using trimethylaluminum [TMAl] and diethylzinc [DEZn] which were used as metal precursors for Zn and Al, respectively. Metal precursors and H_2_O were introduced into the growth chamber separately. A high-purity N_2 _purge was also introduced after each metal precursor to remove the residues and by-products. Optimized AZO properties were achieved with a DEZn/TMAl cycle ratio of 19:1 [23]. Under optimal reaction conditions, the growth rate of the ZnO films and that of the Al_2_O_3 _films were 1.5 to approximately 1.6 Å/cycle and about 0.9 Å/cycle in the substrate temperature range of 200°C, respectively. Then, ZnO ARC was synthesized using two-step methods corresponding to the formation of as-prepared AZO seed layers and the growth of nanorods. The precursors used for ZnO synthesis are zinc nitrate (99.99% purity; Sigma-Aldrich Company, St. Louis, MO, USA) and hexamethylenetetramine [HMT] (C_6_H_12_N_4_). The substrates were placed in a heated solution (25 mM) of zinc nitrate and HMT held for 3 h at 85°C. At the end of the growth period, the sample was removed from the solution and immediately rinsed with DIW to remove residuals from the surface.

### Solar cell fabrication

A p-n junction was prepared by a solution processable SOD technique. To produce an n-type region, the phosphorus-doped SOD solution (P509; Filmtronics Inc., Butler, PA, USA) was spin-coated onto a dummy wafer, and the sample was loaded in a conventional quartz-tube furnace at 1,050°C for 5 min, while the target samples were kept at a closely spaced distance. Phosphorosilica film was removed simply by immersing the prepared specimens in buffer oxide etchant for 10 min. The active area of all devices was defined as 1 cm^2^. Indium/gallium eutectic metal (Ga (75%) In (25%) by weight; the melting point, approximately 15.5°C) was used to form an electrical contact on both sides. Notably, the front contact was made using eutectic liquid metals with a gold probe tip on top of a wire array.

### Characterization

The morphological properties of all the samples were characterized by scanning electron microscopy [SEM] (Hitachi S-4800; Hitachi, Ltd., Chiyoda, Tokyo, Japan), and secondary electron [SE] imaging of the cleaved SiMW arrays were prepared with a focused ion beam [FIB] (Seiko SMI-3050SE; Seiko Instruments Inc., Chiba, Japan). X-ray diffraction [XRD] (Rigaku D/MAX 2200H; Rigaku Corporation, Tokyo, Japan) was used to obtain crystallographic structures. The optical properties of the fabricated wire arrays were measured from the ultraviolet to the infrared region using a spectrophotometer (Cary 500; Varian Inc., Cary, NC, USA). Current-voltage measurements were carried out with a source meter (model 2400; Keithley Instruments Inc., Cleveland, OH, USA) and with a Newport 91192 solar simulator system (Newport Corporation, Irvine, CA, USA) (equipped with 1 kW Xenon arc lamp from Oriel). The light intensity was adjusted to simulated air mass [AM] 1.5 radiation at 100 mW/cm^2 ^with a radiant power energy meter (model 70260; Oriel Instruments, Irvine, CA).

## Results and discussion

**Figure 1:**
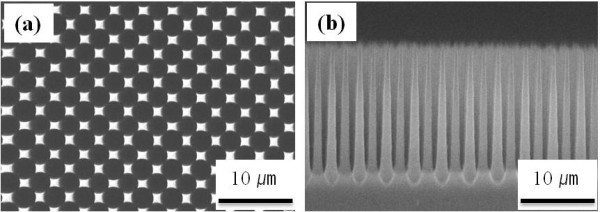
**SEM images of the SiMW arrays fabricated by electrochemical etching**. (**a**) Top view and (**b**) cross section view.

Figure 1 shows the SEM images of the SiMW arrays formed by electrochemical etching. The etching method used in this work to produce ordered arrays of the SiMW is based on the formation of porous silicon using anodic oxidation [24]. By etching a bulk Si substrate in HF electrolyte with electric field, Si can be etched to produce long, straight-walled, uniform pores having micrometer-sized dimensions. The pore formation in p-type Si is believed to occur through a hole-limited silicon dissolution process [24]. As the applied current density increased, the macropores are gradually grown and became interconnected. Finally, a well-ordered array of vertical SiMWs with diamond shape appeared at the corners between the four nearest pores (Figure 1a). Full fabrication process of ordered macropores in p-type Si using HF-based solution is definitely described elsewhere [25,26]. The pore diameter and spacing can be controlled by the current density of the etching, the applied voltage, and the doping of the sample, while the pore length can be controlled independently by adjusting the etching time. The cross section SEM image reveals that the size of the SiMW is 1.5 μm in diameter and 16 μm in length as shown in Figure 1b.

**Figure 2:**
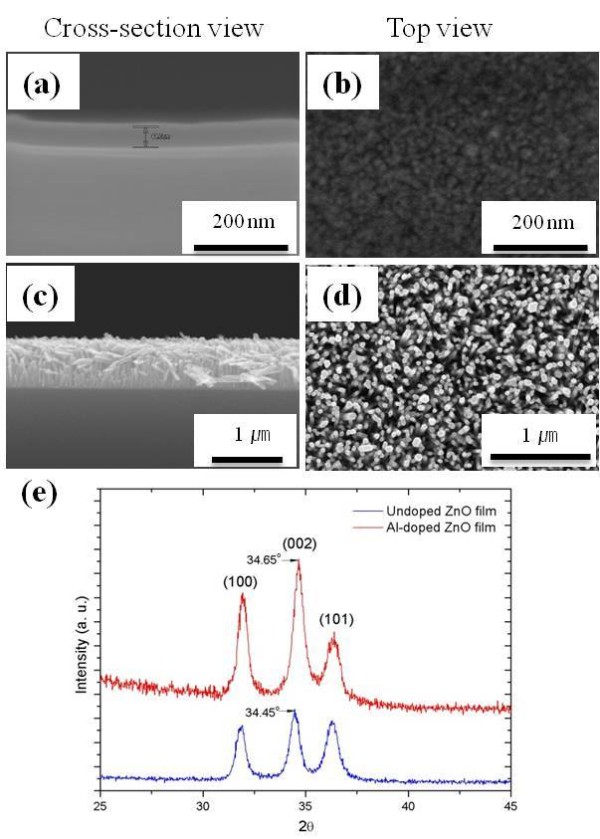
**SEM images of the AZO seed layer and ZnO nanorods**. (**a**, **b**) The AZO seed layer was grown on bare Si and (**c**, **d**) ZnO nanorods were grown on AZO seed layer. (**e**) X-ray diffraction patterns of Al-doped and undoped ZnO films grown on bare Si by ALD.

We have synthesized vertically aligned ZnO nanorods with solution methods using two-step procedures. AZO seed layers were deposited with the ALD system followed by the hydrothermal growth of the ZnO nanorods. Figure 2 shows the SEM images of the AZO seed layer and ZnO nanorods using the AZO seed layer grown on a bare Si wafer, respectively. The thickness of the AZO thin layer is approximately 45 nm (Figure 2a). It can be seen that the AZO films with uniform crystal grain size and flat surface morphology can be obtained by the ALD setup. The arrays consisted of ZnO nanorods with diameters of 50 nm, and the lengths of the rods ranged from 800 nm to 900 nm after the growth time of 3 h by hydrothermal synthesis. Panels c and d from Figure 2 depict that the ZnO nanorods were well grown on Si surfaces that permitted vertically aligned nanocrystal growth to the *c*-axis direction. We also observed that the size of the ZnO nanorods was uniform, and the hexagonal ZnO nanorods demonstrated good crystallinity. The XRD spectra of Al-doped and undoped ZnO thin films grown on Si (100) by ALD are shown in Figure 2e. The major peaks of the ALD-deposited ZnO film appearing at 2*θ *= 31.87°, 34.45°, and 36.31° were assigned to the (100), (002), and (101) planes of the hexagonal wurzite ZnO phase, respectively. It is clear that the preferred growth direction of the undoped ZnO sample is ZnO (002) at a 2*θ *peak of approximately 34.45°. After Al doping, a 0.2° peak shift in a diffraction angle of 34.45° from the wurtzite structure to higher values is observed, which is due to the substitution of Al^3+ ^ions for Zn^2+ ^ions in the ZnO lattice during the growth [27]. The ionic radius of Al^3+ ^cation is 0.54 Å, which is smaller than that of Zn^2+ ^cation (0.74 Å). The substitutional doping of Al^3+ ^at the Zn^2+ ^site will lead to a reduction of the lattice parameter in the ZnO phase and then result in the peak shift [28]. More detailed results and discussions of ALD-deposited AZO films are described elsewhere [29].

**Figure 3:**
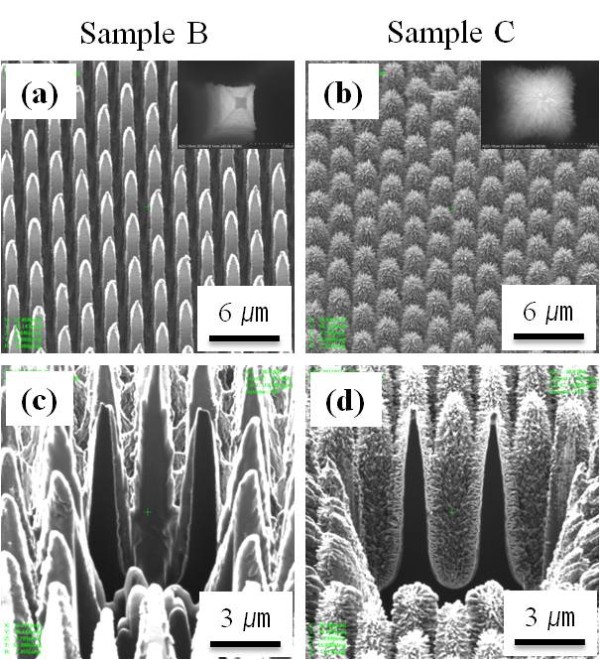
**FIB SE images of sample B and sample C**. (**a**, **b**) Before ion beam sputtering and (**c**, **d**) after ion beam sputtering. The insets in figure manifest the high-magnification image of the single SiMW of each sample.

To investigate the effects of the AZO seed layer and ZnO nanorods on solar cell performances, we have prepared three types of SiMW solar cells. Each sample was defined as SiMW solar cells (sample A), SiMW solar cells with AZO seed layer (sample B), and SiMW solar cells with ZnO nanorods ARC on AZO seed layer (sample C), respectively. First of all, sample A was fabricated by n-type thermal doping on p-type SiMW arrays as demonstrated in Figure 1, and 45-nm-thick AZO thin films were conformally deposited on sample A in order to make sample B. Then, to study the properties of the ZnO nanorods as an AR layer, ZnO nanorods were incorporated on the prepared sample B using hydrothermal growth. We grew vertically aligned ZnO nanorods entirely on SiMW structures and the bottom area of the Si surface. Figure 3 shows FIB SE images of sample B and sample C. As shown in Figure 3, there are observable changes between sample B and sample C. After film coating on sample A, the morphology of the resultant AZO films could not be distinguished by SEM imaging, as shown in Figure 3a. To observe the detailed morphology of the deposited films, cross-sectional FIB SE images of sample B were obtained by the gallium (Ga^+^) primary ion beam sputtering and are shown in Figure 3c. It can be observed that the AZO film thickness was very similar in both side regions of the SiMWs, where the thickness of the AZO film was about 45 nm. This indicates that the highly conformal AZO films were successfully coated on sample A by ALD. In addition, the ZnO nanorod arrays were directed normally along the SiMW surface just the same as those grown on flat bare Si substrates by using the AZO seed layer (Figure 3b). It means that the AZO thin film plays the role of a metal catalyst in VLS as it serves as a buffer layer enabling the nucleation and alignment of ZnO nanorods [20-22]. The results further confirm that the AZO seed layer was uniformly deposited on SiMW solar cells from top to bottom areas. The FIB cross section SE image of sample C after ion beam sputtering is also given in Figure 3d.

**Figure 4:**
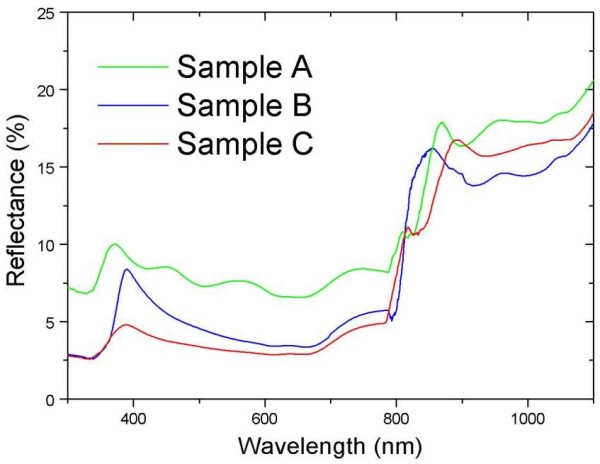
**The reflectance spectra of sample A, sample B, and sample C**.

In order to study the optical properties of all samples, reflectance measurements were carried out using an integrating sphere as shown in Figure 4. The results demonstrate that the reflectance spectrum of sample B was clearly lower than that of sample A over the wavelength range from ultraviolet to the near infrared region. From the above studies, it is believed that the AZO film can be used as a good antireflection coating material [30]. Moreover, in the case of sample C, incorporating ZnO nanorods on sample B, the reflectance values were further decreased in the visible spectrum range. We suggest that the ZnO nanorods trap light, leading to the suppression of light reflection and to the increase in light transmission to the SiMW solar cells.

**Figure 5:**
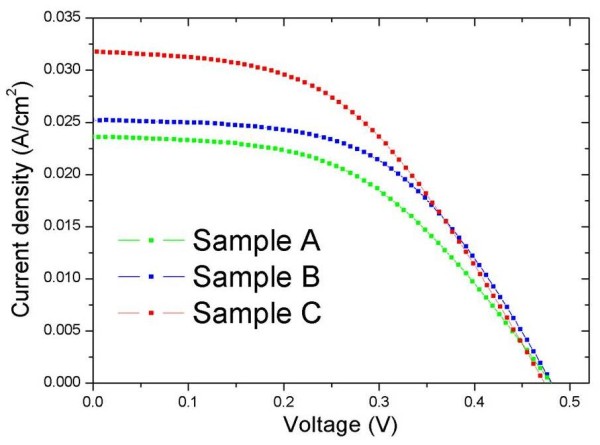
**The light current *J*-*V *characteristics of sample A, sample B, and sample C**.

Finally, the current density-voltage [*J*-*V*] characteristics of all samples were examined in the illuminated condition under 100 mW/cm^2 ^of simulated AM 1.5 global. We found that good agreement between reflectance spectra and cell performances can be achieved through an introduction of the AZO film and ZnO ARC. From the *J*-*V *curves depicted in Figure 5, we observed that the cell performances were drastically improved by introducing the AZO films and ZnO nanorods on SiMW solar cells. Among them, sample C yielded the best performance with an open circuit voltage [*V*_oc_] of 473 mV, a short circuit current [*J*_sc_] of 31.1 mA/cm^2^, a fill factor [FF] of 47%, and a cell efficiency of 7.1%. Comparing sample A with sample B, the light conversion efficiency was increased from 5.6% to 6.4%, indicating an approximately 14% improvement of the total energy conversion efficiency. There are clear improvements in the *J*_sc _and FF. These are mainly caused by the reduced light reflectance and expected reduction in the surface recombination center by AZO coating [31]. Also, from the results of sample B and sample C, we observed that the light conversion efficiency was improved by over 11% and that *J*_sc _was increased by more than 23%. They are attributed to the enhanced light absorption caused by multiple scattering in the ZnO nanorod arrays. Table 1 summarizes the photovoltaic performance of the evaluated samples.

**Table 1:** The photovoltaic performances of sample A, sample B, and sample C

Samples	*V*_oc _(mV)	*J*_sc _(mA/cm^2^)	FF (%)	*η *(%)
Sample A	480	23.6	49	5.6
Sample B	480	25.2	53	6.4
Sample C	473	31.1	47	7.1

## Conclusions

We have fabricated the SiMW solar cells having ZnO nanorod ARC grown on the AZO seed layer and characterized their optical and photovoltaic properties. The morphological results showed that the AZO seed layer and ZnO nanorods were conformally grown on electrochemically prepared SiMW arrays. The combination of the AZO film and ZnO nanorods on SiMW solar cells exhibits the best optical and photovoltaic performances. The photovoltaic efficiency of sample C was enhanced more than 27%, and *J*_sc _was improved by over 31% compared to sample A. It is strongly attributed to the incorporation of the AZO thin film and ZnO nanorod ARC by suppressing light reflectance and surface recombination. The hybrid structures, i.e., SiMW solar cells with transparent conducting oxides, are a promising alternative for efficient energy-harvesting devices.

## Abbreviations

Al: aluminum; ALD: atomic layer deposition; AM: air mass; ARC: antireflection coating; AZO: Al-doped ZnO; c-Si: crystalline silicon; CVD: chemical vapor deposition; DEZn: diethylzinc; DIW: deionized water; FF: fill factor; FIB: focused ion beam; HF: hydrofluoric; HMT: hexamethylenetetramine; *J*_sc_: short circuit current; RIE: reactive ion etching; SEM: scanning electron microscopy; SIMW: Si microwire; SOD: spin-on dopant; TMAl: trimethylaluminum; VLS: vapor-liquid-solid; V_oc_: open circuit voltage; XRD: X-ray diffraction.

## Competing interests

The authors declare that they have no competing interests.

## Authors' contributions

SHB and JHK conceived of the study and participated in its design and coordination. SHB carried out the fabrication and characterization of Si wire solar cells. BYN and IKP carried out the synthesis and characterization of ZnO nanorods. SHB and JHK interpreted together the results and prepared the manuscript. All authors read and approved the final manuscript.
